# Feature Reviews in Cell Death

**DOI:** 10.3390/biomedicines14091905

**Published:** 2026-08-26

**Authors:** Adrian-Bogdan Țigu, Mădălina Nistor

**Affiliations:** Department of Personalized Medicine and Rare Diseases, MEDFUTURE—Institute for Biomedical Research, “Iuliu Hatieganu” University of Medicine and Pharmacy, 400349 Cluj-Napoca, Romania

The term “apoptosis” was introduced in 1972 [1], and nowadays our understanding of how cells die has expanded from a single, immunologically silent program into a rich landscape of genetically encoded, tightly regulated death mechanisms. Necroptosis [2], pyroptosis [3], ferroptosis [4], cuproptosis [5] and disulfidoptosis [6] have joined apoptosis and autophagy-dependent death as distinct execution pathways, each with its own molecular machinery, triggering conditions, and physiological consequences [7]. Regulated cell death sits at the crossroads of oncology, neurodegeneration, infection, and inflammation, and the therapeutic promise of either provoking death in malignant cells or restraining it in vulnerable tissues, has made this one of the most dynamic research areas of contemporary biomedical field.

The “Feature Reviews in Cell Death” Special Issue was conceived to capture that momentum. It brings together six contributions that together span the conceptual breadth of the field: from the molecular anatomy of newly discovered death pathways, through the exploration of cell death in cancer therapy, to the pathological role of neuronal loss in neurodegenerative disease. Read as a collection, these articles illustrate a central theme of modern cell death research: the same core machinery that safeguards tissue homeostasis can be harnessed as a therapeutic weapon or must be defended against as a driver of disease.

**Defining the newest death pathways**

A recurring lesson of the past decade is that cell death remains an actively expanding field. Wand et al. [6] provide a timely and thorough synthesis of disulfidoptosis, a form of regulated cell death, first described only recently [8], in which cancer cells overexpressing the cystine transporter SLC7A11 accumulate excessive disulfide bonds under glucose deprivation, collapsing the actin cytoskeleton and triggering a death program mechanistically distinct from apoptosis or ferroptosis. Their review maps the molecular basis, regulatory networks and pharmacological inhibitors of disulfidoptosis, and carefully situates it relative to the other regulated cell death modalities, before turning to its emerging relevance in tumors and chronic diseases.

**Cell death as a therapeutic lever in cancer**

Through the lens of tumor metabolism, Jiang et al. [9] analyze immunogenic cell death (ICD). The process of ICD transforms dying cancer cells into a source of numerous damage-associated molecular patterns (DAMPs), including calreticulin, HMGB1, etc. [10,11]. The authors analyze the impact metabolic switch, such as the Warburg effect [12], oxidative stress, and abnormal lipid metabolism, has on the efficacy of ICD and the structure of the immune microenvironment. The authors connect mitochondrial and endoplasmic reticular stress and ferroptosis to immune activation and analyze treatment methods. The paper is a useful bridge between two fields, metabolism and immuno-oncology, that are increasingly studied together.

Markov et al. [13] focus on sesquiterpene lactones (STLs) as potential agents to treat glioblastoma. Known as one of the deadliest cancers, glioblastoma is characterized by invasive growth and resistance to radiation and chemotherapy [14]. Summarizing ten years of research, it is shown that STLs can penetrate the blood–brain barrier and have a multi-target effect by participating in mitochondrial dysfunction, oxidative stress, and apoptosis as well as inhibiting glucose metabolism and cell cycle progression and suppressing proneural-mesenchymal transition. This ability to kill tumor cells and to stop their spreading makes natural compounds very attractive in a disease that has seen many single-target medications fail.

Tigu et al. [15] reviews the epigenetic therapies in melanoma, focusing on DNA methylation and histone modification. Against a backdrop of rising melanoma incidence [16], they highlight that reversible epigenetic alterations represent an attractive and in principle druggable axis of tumor biology. Drug repositioning emerges as a pragmatic strategy for accelerating clinical transition.

**Cell death beyond oncology, a focus on infections and neurodegeneration**

Werawatganone et al. [17] discuss the efficacy of the *Gardenia jasminoides* fruit extract in addressing the issues of *Helicobacter pylori*-induced gastritis. Through a combination of in vitro studies and experiments performed on Sprague–Dawley rats, the authors found that the extract abundant in geniposide suppresses the growth of *H. pylori*, stabilizes the membranes of red blood cells, which indicates the anti-inflammatory effect of the extract, and inhibits the increase in the levels of inflammatory agents such as IL-17, IL-33, and gastric EGF known to rise in the course of histopathological changes in the stomach. The findings illustrate the impact of natural products on the inflammation-related tissue damage and cell death resulting from chronic infections.

According to Kwak et al. [18], regulated cell death is a pivotal point in understanding Alzheimer’s disease. Instead of attributing neuronal loss to any specific mechanism, they provide evidence from human samples, animal models, and in vitro, indicating that amyloid β, tau pathology, oxidative stress, and chronic neuroinflammation are involved in the activation of apoptosis, necroptosis, pyroptosis, ferroptosis, and autophagy, with activity varying by time and cell type. Connecting these molecular patterns to the recent immunohistological, imaging, and biofluids methods of diagnosis shows the avenues of path-specific biomarkers and complex neuroprotective approaches. Thus, in this case, the therapeutic goal in contrast to oncology is not death but its prevention.

**Concluding remarks**

These six articles outline the dual nature of cellular death in human disease. In the case of cancer, one must aim at deliberately inducing death: immunogenicity, susceptibility to metabolism, epigenetics, or other novel processes can be used for achieving this goal, such as disulfidoptosis. Yet, in neurodegenerative diseases and chronic inflammation, individuals aim to inhibit the mechanisms of cellular death to preserve the vulnerable tissue. The unifying theme emerging here is the research approach used, implying multi-target and combinatorial therapies, drug repurposing, natural agent pharmacology, and the use of molecular mechanisms in combination with clinical measures.
